# Healthy gut microbiomes are host-controllable microbiomes

**DOI:** 10.3389/fmicb.2024.1497083

**Published:** 2025-01-08

**Authors:** Théodore Bouchez, Bin Liu, Daniel Rios Garza

**Affiliations:** ^1^Université Paris-Saclay, Institut national de recherche pour l'agriculture, l'alimentation et l'environnement (INRAE), PRocédés biOtechnologiques au Service de l'Environnement, Antony, France; ^2^Key Laboratory of Environmental Biotechnology, Research Center for Eco-Environmental Sciences, Chinese Academy of Sciences, Beijing, China

**Keywords:** microbiome, control theory, microbiome ecology and evolution, dysbiosis, gut microbiome

## Introduction

“The instinct of each species is good for itself but has never, as far as we can judge, been produced for the exclusive good of others” (Darwin, 1859). Why, then, do humans and other mammals benefit from their microbes?

Evolutionary theory predicts that natural selection can act at organizational levels higher than the gene or individual—such as on groups or even species—provided that the selective advantages for the group outweigh evolutionary conflicts. Darwin suggested that natural selection can act “on the family, and not the individual,” “for the sake of gaining a serviceable end” (Darwin, 1859). This idea was later formalized as kin selection (Hamilton, 1964). Even distantly related organisms can align their evolutionary goals, as demonstrated by the longstanding partnership between eukaryotic cells and mitochondria (Margulis, 1970).

Microbes are an integral part of animal and plant hosts, fulfilling essential physiological roles, such as enabling access to otherwise unavailable nutrients, training the immune system, supporting mucosal development, and providing protection. Some microbes are even vertically transmitted from parents to offspring. The numerous examples of microbes performing vital functions for their macrobial hosts makes it tempting to assume that humans and the microbes residing on and within our bodies—particularly in our guts—evolved to share common selective interests that outweigh potential evolutionary conflicts (Rosenberg and Zilber-Rosenberg, 2013; Zilber-Rosenberg and Rosenberg, 2008; Gilbert et al., 2012; Malard et al., 2021). Supporting this assumption, several reports suggest that humans and other mammals are selected for harboring “good” microbes and that these “good” microbes thrive by helping their hosts, for example, by contributing over 95% of our organism's genetic repertoire (Malard et al., 2021; Grice and Segre, 2012; Martino et al., 2022). But is group-level selection a necessary and sufficient mechanism to explain why microbiomes benefit their hosts?

Microbes certainly influence a host's chances of survival and reproductive success (Yuval, 2017; Gould et al., 2018) but the reasons why microbes would reduce their own fitness to form an evolutionarily aligned group with their hosts and other microbial populations—an essential condition for group-level selection—are not easily explained (Douglas and Werren, 2016; van Vliet and Doebeli, 2019). For example, during a 25-year human generation, gut microbes undergo more than 50,000 generations (assuming a conservative 4-h generation time), competing with thousands of microbial species. Roughly half of the bacterial biomass in the colon is lost daily and replaced by new bacterial growth (Arnoldini et al., 2018; Stephen and Cummings, 1980). Before a human reproduces, any trait unfavorable to a microbe's growth or survival would likely be eliminated. Moreover, the human gut is a relatively open system, regularly exposed to diverse environmental microbes. Vertical transmission accounts for only a small fraction of the microbiome. About 20 species are consistently shared among most adults (Qin et al., 2010). How, then, has evolution shaped such a diverse, rapidly evolving microbial ecosystem to consistently benefit individual health?

One might argue that by providing advantages to “good” microbes, the host mitigates evolutionary conflicts, encouraging microbes to sacrifice some fitness for the host's benefit. However, as microbes rapidly evolve, new genotypes with similar needs can emerge to exploit these host-provided advantages without incurring costs—an example of the tragedy of the commons (Hardin, 1968). These benefits are shared among all compatible microbes, whether or not they contribute to the host's wellbeing, creating an imbalance between contributors and free riders. A free rider, for example, could be a bacterium that feeds on mucin glycans provided by the host while invading tissue and potentially causing severe illness.

Why, then, do humans and other mammals benefit from their microbes? A recent model by Sharp and Foster (2022) suggests that one way to overcome this evolutionary conundrum is for hosts to evolve mechanisms to actively control their microbes and limit the opportunities for microbes to evade control. This would require mechanisms of enforcement or policing to maintain cooperation (Ågren et al., 2019). In this view, hosts are selected not for harboring “beneficial” microbes but for their ability to maintain their microbiomes in a healthy state. In other words, evolution might favor hosts as microbiome control engineers—or, more accurately, control tinkerers (Jacob, 1977)—who make use of various control methods within reach of the evolutionary landscape to sustain their microbiomes in a healthy state. These include responses ranging from rudimentary mechanisms such as inflammation or diarrhea to the sophisticated responses driven by the adaptive immune system. From this perspective, control theory—the study of how systems regulate themselves to achieve desired outcomes—parallels host-microbial interactions, suggesting that healthy microbiomes might be, by definition, host-controllable microbiomes.

## Will a large complex microbiome be stable?

**Figure 1:**
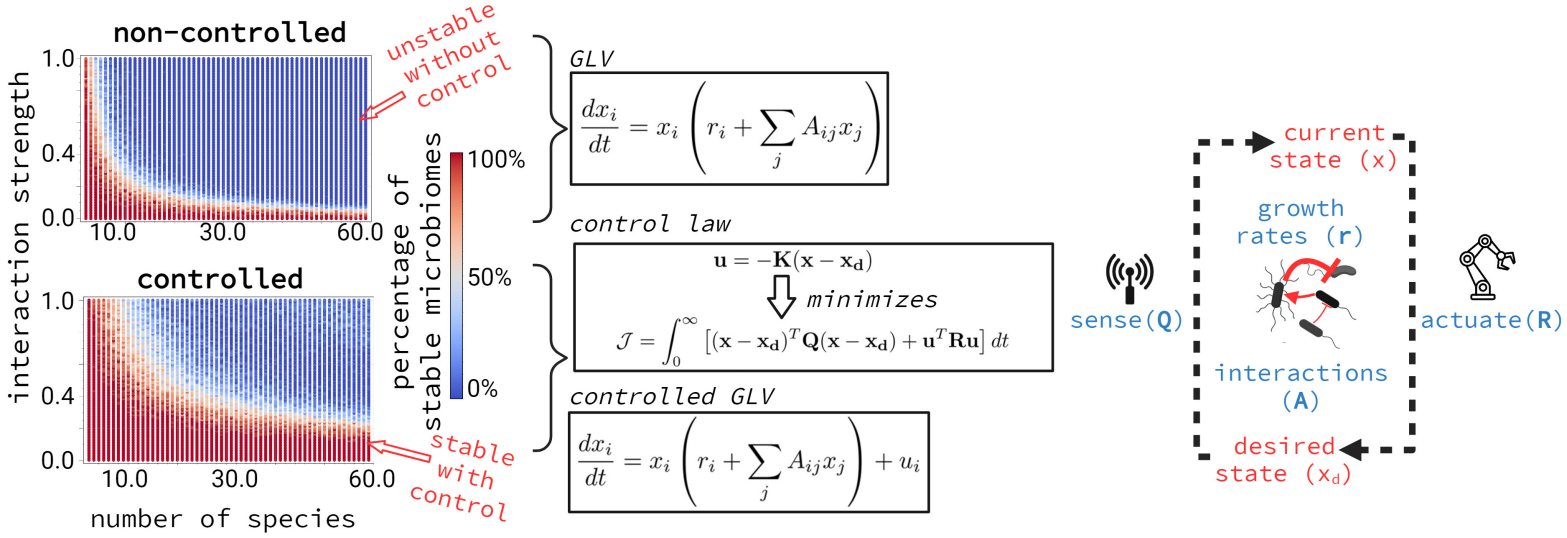
Fraction of stable microbiomes with and without a controller in 10,000 GLV simulations. Random microbiomes were simulated with increasing interaction strengths and numbers of species. Each dot represents 100 simulations and is colored according to the fraction that resulted in a stable microbiome. Dark blue dots indicate cases where all 100 simulations were unstable, while dark red dots indicate cases where all were stable. The white transition zone represents intermediate stability. The results show that adding a controller—here idealized as a device that senses the microbiome's current composition and adjusts species growth rates to guide the system toward a desired composition—significantly increases the range of stable microbiomes. Stability, as defined here following May's original model, refers to whether the system returns to equilibrium after a small perturbation based on the signs of the eigenvalues of the Jacobian matrix at the equilibrium point.

In the 1970s, writing in *Nature*, Robert May proposed a limit to the stability of ecosystems as complexity increases, particularly as the number and strength of species interactions grow (May, 1972). May analyzed a simplified Generalized Lotka-Volterra (GLV) model to explore this concept. The model simulates random communities where population sizes are constrained by resource availability (represented as negative self-interactions) and by mutual positive or negative influences on one another's growth. According to the GLV model, assembled communities are predicted to remain stable only when interactions are sufficiently weak or when strong interactions are confined to a few species (Figure 1).

GLV-like models are commonly applied to the study of microbiomes, where similar stability/complexity trade-offs are assumed to hold (Coyte et al., 2015; Yonatan et al., 2022). However, even in May's simplified model, introducing a controller—an agent that directs the system toward a predetermined objective, such as through energy expenditure—is one way to continuously stabilize an otherwise unstable system and reshape the stability-complexity landscape (Figure 1).

Increased microbiome diversity is generally associated with a healthy gut. For example, patients suffering from pathogen invasions, gut inflammation, colitis, inflammatory bowel disease, or colorectal cancer show reduced alpha diversity compared to healthy controls. Similarly, factors such as aging, obesity, the Western diet, and urbanization are also linked to a decline in microbiome diversity (Le Chatelier et al., 2013). Diversity is not necessarily the cause of a healthy microbiome, but it could rather be a consequence of and indirect evidence for host control. In other words, host control effectively drives the microbiome toward a healthy state, which, in the gut microbiome, is also a more diverse state than would occur in the absence of control (due, for example, to stability constraints). In this case, when control is impaired, diversity also declines.

In other biological systems, active control mechanisms can instead reduce microbial diversity. Some examples are laboratory techniques like selective media and dilution-to-extinction procedures, which isolate single strains, or environmental factors such as antibiotics, physical barriers, extreme pH, or heat conditions that similarly reduce microbial diversity. In nature, we see examples of active control reducing diversity, such as the Hawaiian bobtail squid, which isolates a single species of Vibrio fischeri from the ocean in its light organ (Wollenberg and Ruby, 2009). However, it is less often appreciated that active control can also promote greater diversity than would be stable in the absence of control.

An intriguing and underexplored perspective is that the host actively controls the microbiome's natural diversity and that declining diversity may signal a loss of controllability, as suggested by this simplified model (Figure 1). For instance, microbiomes established in reactors that simulate the gastrointestinal tract are significantly less diverse than their original stool inocula (Chassaing et al., 2017). Although these reactors replicate the physicochemical conditions and flow rates of the gut, they lack host-control mechanisms, such as the immune system and dynamically regulated environmental factors. Overall, viewing the gut microbiome as an interconnected, island-like ecosystem—where each individual serves as a microbial patch colonized by dispersal, local diversification, environmental selection, and drift (Costello et al., 2012)—becomes clearer when we consider environmental selection processes as active, energy-expending efforts by the host to target desired diversity, composition, and functions.

Beyond diversity, the host's control also extends to the microbiome's “state-space,” which includes microbial populations and their biochemical activities. In an ideal scenario of tight, full-state control, the host would control both the abundance and function of each microbial population, including their chemical outputs and inputs. However, a more realistic scenario involves limited, partial-state control, where the host exerts varying degrees of influence over distinct aspects of the state-space. For example, a recent theoretical framework proposed that the composition of the gut microbiome can be controlled by targeting specific sets of microbes, referred to as “minimal control elements” (Angulo et al., 2019). This control can be achieved by leveraging species-species interactions, which form control chains connecting external controllers to the intrinsic dynamics of community interactions (Angulo et al., 2019). Although this framework still lacks empirical validation, it highlights the potential for identifying specific control elements to restore a dysbiotic microbiome.

## How control theory helps explain microbiomes

The mammalian gut contains various active control mechanisms (Wilde et al., 2024; Foster et al., 2017), which can be categorized into microbiome-independent and microbiome-dependent processes. For example, transit time ranges from 2 to 5 h in the small intestine and 10 to 59 h in the large intestine (Lee et al., 2014). These parameters directly influence the microbiome—favoring host absorption over microbial growth in the small intestine and microbial growth and fermentation in the large intestine—but are generally independent of sensing microbiome features. From the microbiome control perspective, these processes are open-loop controllers (Brunton and Kutz, 2022)—that is, they operate without feedback from the microbiome itself. By contrast, the immune system's targeting of specific microbiome features exemplifies a closed-loop controller, which adjusts its actions based on feedback from microbial dynamics.

Evolving closed-loop microbiome controllers likely allowed humans and other mammals to fine-tune their responses and maximize their benefits from large, diverse, and densely populated microbiomes. For example, a meta-study comparing cultures of fresh stool samples in reactors that, to varying degrees, model the gut environment (e.g., SHIME, TIM-2) found that interindividual variation in the inoculum is the primary driver of community composition (Garcia Mendez et al., 2024)—i.e., interindividual variation outweighs the selective pressure exerted by the reactor's operational design (biotope). The evolution of closed-loop control likely ensures that these widely different, personalized microbial communities, assembled by each individual over their lifetime, continue to perform essential functions despite their variability.

## Controllability and observability are fundamental limit conditions for microbiome control

If microbiome control is necessary for the host to maintain a healthy state, then we need models and experimental systems to elucidate two essential limit conditions for microbiome control: observability (which aspects of the state-space can the host observe?) and controllability (which states can the host influence through control inputs, ideally within a finite time?). Numerous studies associate high consumption of simple sugars and low dietary fiber intake with dysbiotic microbiomes. However, the underlying mechanisms remain widely debated. One possible explanation is the loss of both controllability and observability.

Experiments with synthetic microbiomes suggest that complex substrates result in history dependence, where microbiomes diverge in taxonomic composition and function (Bittleston et al., 2020; Leventhal et al., 2018; Silverstein et al., 2024). In contrast, simple substrates lead to more deterministic compositions, particularly at higher taxonomic levels (Goldford et al., 2018). Put simply, multiple states are possible for microbiomes grown on complex substrates, even when isolated from the same source and maintained under identical conditions (Leventhal et al., 2018). A subset of these states in the gut may correspond to healthy microbial communities. The host's control mechanisms could guide the microbiome toward these healthy states.

In contrast, synthetic communities grown on simple substrates tend to converge to similar taxonomic compositions and metabolic profiles, regardless of their sources (Goldford et al., 2018; Estrela et al., 2022). In this scenario, the host's control may be limited, as healthy microbiome states could be outside its reach. For example, patients with low microbiome richness often respond poorly to therapeutic interventions, including dietary restriction and cancer immunotherapy (Routy et al., 2018).

Controllability is inherently dependent on observability. Specifically, controlled colonization events educate the immune system to recognize pathogens and enhance the host's ability to monitor microbial states. For instance, *Bacteroides fragilis* produces an immunomodulatory polysaccharide that, in germ-free mice, trains a population of immune cells to protect against *Helicobacter hepaticus*, a pathogen responsible for colitis in immunocompromised animals (Mazmanian et al., 2005, 2008). Another example is segmented filamentous bacteria (SFB) (*Candidatus Arthromitus*), a species-specific filamentous bacteria found in the guts of several vertebrates. SFB forms millimeter-long filaments that attach directly to the ileal epithelium via a specialized cell called a holdfast, without triggering inflammation (Schnupf et al., 2015). In several species, SFB colonization peaks during early life (Hedblom et al., 2018), training the immune system against future invasive pathogens.

Interestingly, SFB occupies a spatial gradient similar to environmental cable bacteria, connecting the oxygen-rich epithelium to the anoxic lumen. Although they share similar morphology—long, single-layered filaments—it remains to be tested whether SFB plays a similar ecological role in conducting electrons between these zones (Nielsen et al., 2010) and sharing them with other species (Bjerg et al., 2023). Ericsson and colleagues observed that only the stool of mice harboring SFB produced a strong exoelectrogenic current in microbial fuel cells, even though the SFB itself was not found on the electrodes (Ericsson et al., 2015). Their study also showed that differences in the electrical current production by exoelectrogens were predictive of lymphocyte trafficking to the gut. In other words, the mammalian gut may monitor and respond to the unique electrical signatures of its microbiome, targeting microbes at locations with unusual signatures. By adapting its immune response to specific electrical signatures or redox gradients, the host may enhance its ability to observe and react to changes in the microbiome's state-space.

## Host-microbiome dynamics: Pinocchio and Geppetto

Overall, control incurs fitness costs for the host, both for gathering information and for acting on that information. From an evolutionary perspective, hosts minimize these costs to invest on growth and reproduction. Over different timescales, an evolutionary arms race develops between hosts and microbial populations: hosts benefit from efficient, cost-effective control strategies, while microbes evolve ways to evade such control—especially when it restricts their survival and growth (Wilde et al., 2024).

For example, inflammation is a systemic response by the host to counter microbial invasion. When triggered, reactive oxygen species are released. Some opportunistic pathogens exploit this response by tolerating the released hydrogen peroxide and expressing molecular machinery that allows them to absorb it into their cytoplasm. There, they quickly convert it into oxygen for use in their respiratory chain, giving them an advantage over fermentative organisms (Crowley and Vallance, 2020). *Salmonella enterica serotype* Typhimurium goes a step further. Under normal conditions, the gut detoxifies hydrogen sulfide, produced in large quantities by the microbiota, by converting it into thiosulfate. During inflammation, reactive species convert thiosulfate into tetrathionate, which *S. Typhimurium* respires (Winter et al., 2010), effectively feeding on the host's defense mechanisms.

A healthy, symbiotic microbiome remains under host control, maintained by the host's regulatory mechanisms to ensure beneficial function. In contrast, a dysbiotic microbiome arises when this control is impaired—whether because a healthy composition becomes unreachable, necessary cues become unobservable, or the mechanisms of control (effectors) become ineffective. In other words, microbes are not our Geppetto (Stilling et al., 2016); instead, we attempt to control them. Like Pinocchio, they strive—and often succeed—to evade our control.
